# Developing Botulinum Toxin Drugs: Unexpected Challenges

**DOI:** 10.1007/s00702-025-03095-8

**Published:** 2026-01-27

**Authors:** Dirk Dressler, Jürgen Frevert

**Affiliations:** 1 Movement Disorders Section, Department of Neurology, Hannover Medical School, Carl-Neuberg-Str. 1, 30625 Hannover, Germany; 2 Neurotoxin Research Center, Tongji University Medical School, Shanghai, China; 3 Merz Pharmaceuticals, Frankfurt/M, Germany

**Keywords:** Botulinum toxin, Drugs, Development, Potency labelling, Specific biologic activity, Immunogenicity

## Abstract

The development of a new botulinum toxin (BT) drug is a major project associated with numerous challenges. Obvious ones include financing, manufacturing, registration and marketing. They will not be covered here. We want to cover challenges, which may be unexpected, but still will be crucial to the success of the development project. Potency is the most important critical quality attribute (CQA) of BT drugs. It is based on biological assays and the manufacturers’ internal reference standards. Clinical experience shows that the potency labelling of BT drugs is not directly comparable. As long as external reference standards do not exist, comparative data on the potency labelling should be provided for all new BT drugs. Idiosyncratic potency labelling will hinder the clinical use and the positioning of a new BT drug in the market. Therapeutic profiles are also a CQA. They describe efficacy and safety of BT drugs. They may be influenced by various molecular differences amongst the different BT types and BT subtypes. Therapeutic profiles can only partly be explored in animal experiments. As extrapolation to a human therapeutic use is problematic, human pilot studies would be helpful. Immunogenicity is another pivotal CQA. It can best be described by the specific biological activity (SBA). In the absence of animal models, the SBA of new BT drugs should be determined to predict immunogenicity, before post-registration studies become available to allow analysis of large number of patients over prolonged periods of exposure. Manufacturers of new BT drugs will make product claims, usually as unique selling points. These claims will be scrutinised by the medical community and challenged by the FDA. If they can’t be substantiated by robust scientific data, they will not be accepted for the registration documents. Undelivered promises may jeopardise the success of the development project.

## Introduction

Botulinum toxin (BT) is used therapeutically since the late 1980s. For many years, BT drugs were provided by a small group of manufacturers including Allergan (onabotulinumtoxinA (ONA, Botox^®^), Ipsen (abobotulinumtoxinA, ABO, Dysport^®^), Elan/US WorldMeds/Solstice/Supernus (rimabotulinumtoxinA, RIMA, MyoBloc^®^, Neurobloc^®^), Merz (incobotulinumtoxinA (INCO, Xeomin^®^) and Lanzhou Institute of Biological Products (lanbotulinumtoxinA, LAN, Hengli^®^). Recently, stimulated by an increase in aesthetic use, the number of BT manufacturers almost exploded, especially in Korea (Dressler et al. in press).

Before a BT product can be registered as a drug, it must pass a complex registration process overseen by various national authorities, including the United States Food and Drug Administration (FDA), the European Medicines Agency (EMA), the Korean Food and Drug Administration and Health Canada. Although much effort has been made to synchronise these processes, differences still exist. Good Manufacturing Practice (GMP) regulations pose a significant challenge for BT drug manufacturing.

For all manufacturers, the development of a new BT drug is a major project associated with numerous challenges. Obvious challenges include financing, manufacturing, registration and marketing. These challenges will not be covered here. Instead, we want to cover challenges, which may be unexpected, but still will be crucial to the success of the development project.

## Potency labelling

Potency is the most important critical quality attribute (CQA) of BT drugs. Potency describes the biological activity of a BT drug. Uncertainties about potency bear substantial risks for BT’s clinical use. They also make comparative studies on efficacy, safety and costs impossible.

Potency of BT drugs is best measured in biological models, typically in lethality assays based on dose-effect curves monitoring mouse lethality. For potency measurements of BT drugs, a standard mouse lethality assay has been described in much detail in various pharmacopoieae with potencies given in LD50 units or mouse units (MU). Although these lethality assays are standardised, clinical practise shows, that the MU determined by them are not identical. As a consequence of this, the FDA has explicitly warned, that the potency labelling of BT drugs are not directly comparable.

Whereas MU of Allergan, Merz and Lanzhou are identical (Dressler 2010; Dressler et al. 2012, 2014, 2018; Pan et al. 2019), Ipsen’s MU are clearly different and a conversion factor needs to be applied. However, there is - so far - no agreement as to which conversion factor might be appropriate (Scaglione 2016). Suggested conversion factors range from 1:1 (Wohlfahrt et al. 1997) to 1:11 (Marchetti et al. 2005). Supernus MU are also idiosyncratic and have to be converted by using a conversion factor of 1:40 to become comparable to MU of ONA, INCO and LAN (Dressler and Eleopra 2006).

The idiosyncrasy of Ipsen’s and Supernus’ potency labelling became only apparent after the registration and after more wide-spread clinical experience was gathered.

For the development of new BT drugs an unambiguous potency labelling is critical. Basing the potency labelling on an external industry standard would have solved this problem. However, manufacturers did not agree on this and still use their internal potency reference standards. As long as internal potency reference standards are applied, conversion factors should be provided by the manufacturers to allow comparisons on efficacy, safety and costs. Reference to the potency labelling of ONA, INCO and LAN would be preferable, whereas idiosyncratic potency labelling will hinder the clinical use and the positioning of a new BT drug in the market.

### Therapeutic profiles of BT types and BT subtypes

Therapeutic profiles describe the efficacy and safety of BT drugs. For efficacy, the onset latency and duration of the therapeutic effect are described and for safety, the kind of adverse effects. They are directly related to BT’s molecular structure. They are also a CQA of BT drugs.

BT is a di-chain protein produced by Clostridium botulinum (Dressler and Foster 2018). BT’s mode of action includes three steps. In the binding step, in which BT binds with its heavy chain to ganglioside acceptors on the neuronal cell surface and co-binds to the specific BT receptors synaptotagmin or synaptic vesicle protein 2 (SV2) depending on the BT type (Dressler and Foster 2018). In the translocation step, BT is internalised into the nerve terminal cytosol. In the cleavage step, BT’s light chain cleaves one or two of the SNARE proteins SNAP25, VAMP (Synaptobrevin) and Syntaxin, again depending on the BT type. This blocks the secretion of acetylcholine into the synaptic cleft (Pantano and Montecucco 2014). 

Although the protein structure of the BT types and BT subtypes is similar, differences do exist. All of those molecular differences may affect all three elements of BT’s mode of action including binding, translocation and SNARE protein cleavage and - with this- may directly affect BT’s therapeutic profile.

For BT type A (BT-A), the therapeutic profile is well established by animal studies and by extensive clinical use covering numerous indications, large patient populations and its prolonged clinical use.

BT type B (BT-B) was initially developed as an alternative to BT-A. As predicted from animal experiments, its efficacy is similar to BT-A with similar onset latency and similar duration of action. Its safety, however, differs substantially from that of BT-A (Dressler and Benecke 2003): whereas BT-A has a relatively strong effect on neuromuscular cholinergic synapses, BT-B has a relatively strong effect on autonomic cholinergic synapses. This means, in order to produce sufficient muscular efficacy, strong autonomic adverse effects have to be accepted. In animal studies, these safety differences were not detected and neither the manufacturer nor the FDA anticipated them, so that they were not monitored in the registration studies. These autonomic adverse effects only became apparent, when larger patient populations were treated and independent and unbiased observations became possible after the drug became widely available in the market (Dressler and Benecke 2003).

BT type E (BT-E) (trenibotulinumtoxinE, TRENI, Allergan-Abbvie) is currently under clinical investigation. Animal studies and clinical data suggest a special therapeutic profile with rapid onset and short duration of action (Yoelin et al. 2018; news.abbvie 2023). The complete safety profile, however, is not yet available, so that potential autonomic adverse effects cannot be evaluated.

For other non-A- and non-B-BT types, animal data only describe some aspects of their efficacy usually durations of action and onset latencies. Human experience is restricted to few experimental BT applications only (Dressler et al. 2019; Eleopra et al. 2020; Chen et al. 1998; Greene and Fahn 1993; Sheean and Lees 1995).

For BT subtypes such as BT subtype A6 (BT-A6) (Moritz et al. 2018; Whitemarsh et al. 2013) only preliminary animal data on efficacy dynamics are available. A current BT-subtype A2 (BT-A2) drug development project provides some preliminary clinical data in addition to animal efficacy dynamics data (Takeuchi et al. 2021).

For BT drug developmen, this means, that animal studies are not sufficient enough to predict therapeutic profiles, neither with respect to efficacy nor with respect to safety. Whereas differences between BT types may be substantial, differences amongst BT subtypes may be minor. Obviously, this bears substantial risks for BT drug development: favourable therapeutic effects predicted by animal experiments may not be confirmed in human applications and adverse effects not detected in animal experiments may occur in human use.

Pilot studies in humans would help to predict therapeutic profiles in patients. Unfortunately, they have become increasingly difficult to perform, as BT study material now has to be drug grade and has to be manufactured according to FDA-GMP standards.

## Immunogenicity

**Fig. 1:**
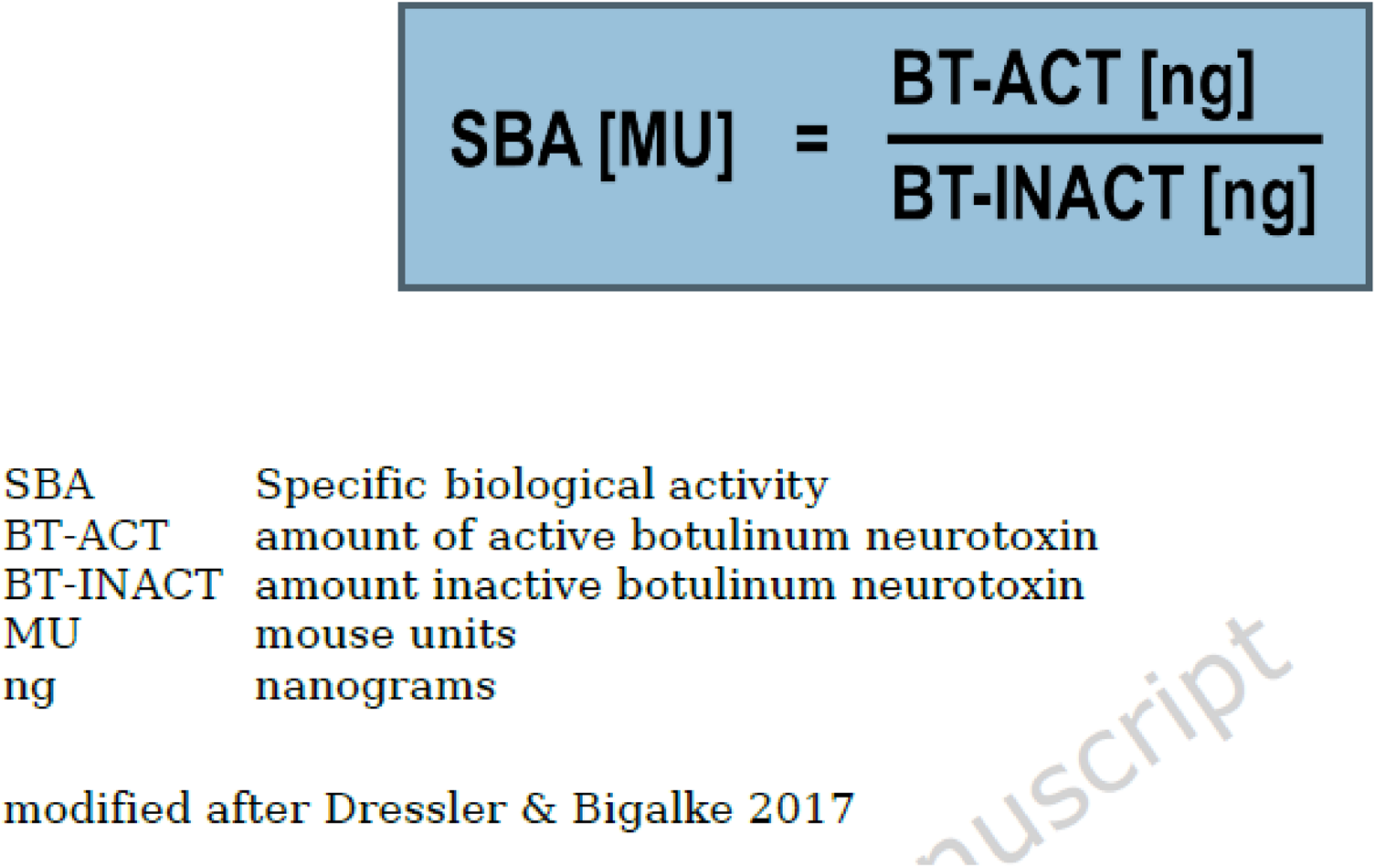
Specific biological activity

Immunogenicity is another CQA of BT drugs. It describes the potency of BT drugs to trigger formation of BT antibodies (BT-AB). As BT is a foreign protein, patients being exposed to BT drugs may develop BT-AB in the course of their BT therapy (Dressler and Bigalke 2017). These BT-AB may reduce or completely block BT’s therapeutic action (Dressler 2004). One of the risk factors for BT-AB formation is the amount of BT applied to the patient (Dressler and Bigalke 2017). The immunologically relevant amount of BT includes not only the biologically active BT (BT-ACT), but also the biologically inactive BT (BT-INACT). BT-ACT produces the therapeutic effect. It is pre-determined by the patient’s condition and its therapeutic requirements. BT-INACT consists of BT inactivated during the manufacturing process and during prolonged storage. It may also consist of un-nicked BT, i.e. BT not activated during the manufacturing process. BT-INACT does not contribute to BT’s therapeutic effect, but still may act as an antigen for BT-AB formation. BT drugs, therefore, may have different immunological qualities depending on the amount of BT-INACT included. This immunological quality of BT drugs was first recognised and described as the specific biological activity (SBA) by Dressler (Dressler and Hallett 2006). As shown in Fig. 1, the SBA describes the relationship between the BT drug’s potency in MU and the BT drug’s total amount of BT in nanogram including BT-ACT and BT-INACT.

**Fig. 2:**
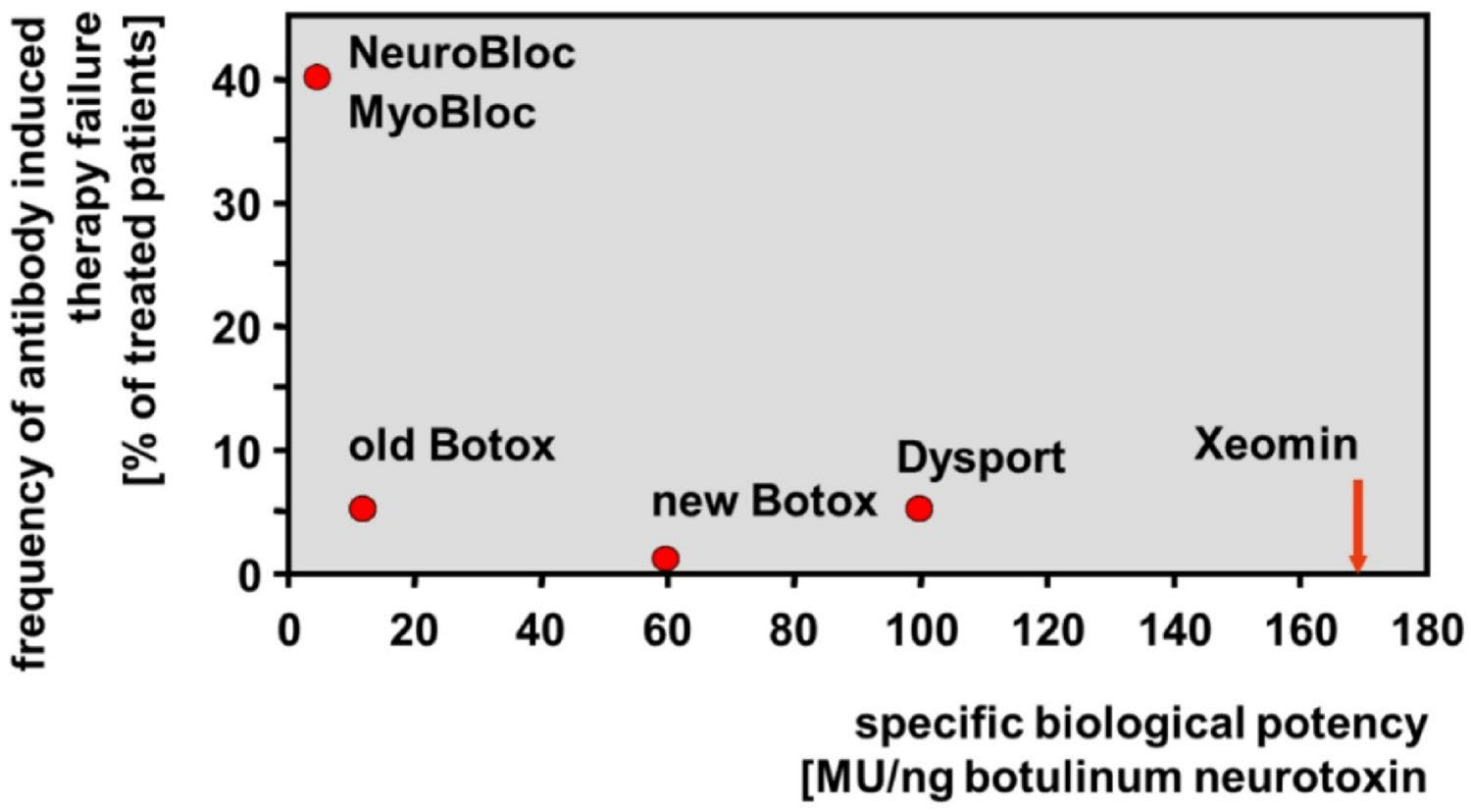
Specific biological potency and frequency of antibody induced therapy failure

Figure 2 shows the correlation between SBA and the frequency of complete antibody-induced BT therapy failure (CABF) in patients treated for cervical dystonia. RIMA stands out with a particular low SBA, which is related to a very high CABF frequency of around 40% (Dressler and Bigalke 2005). This RIMA immunogenicity problem was neither detected by the manufacturer during the drug development, nor by the registration authorities. It was only detected during repeated clinical applications exceeding the two or three BT applications monitored for the registration process (Dressler and Bigalke 2005).

Figure 2 also shows a difference in CABF frequency between ‘old Botox’ and ‘new Botox’. In 2000, ‘new Botox’ replaced the initial version of Botox^®^ based on BT batch 79/11 manufactured by Edward J Schantz and Eric A Johnson in November of 1979 (Racette et al. 1999; Jankovic et al. 2003). The prolonged storage of the original BT batch had caused a gradual decline in potency, so that the SBA dropped (EA Johnson, personal communication). When ‘new Botox^®^’ was introduced, a continuous manufacturing process was introduced and the increased SBA reduced the CABF frequency (Jankovic et al. 2003).

The nature of the drug substance can also influence the formation of antibodies. ONA, ABO and most of the Korean products contain the BT associated with complexing proteins (neurotoxin associated proteins, NAP). These NAP have no therapeutic function, but might stimulate antibody formation (Frevert and Dressler 2010).

Immunogenicity is and will be a major issue of BT therapy, especially as most BT applications are chronic ones requiring prolonged treatments. Therefore, controlling and maintaining current immunological BT drug standards is of major importance. As there are no animal models for immunogenicity testing of BT drugs, SBA evaluation seems advisable. Requests for post-registration data by the registration authorities might also be helpful.

## Product claims

When new BT drugs are developed, the manufacturers will make product claims about special features of their product, usually as unique selling points differentiating their drugs from others. These claims, however, need to be substantiated by scientific data in order to be accepted by the FDA for the registration documents. When the drug development project for daxibotulinumtoxinA (DAXI, Daxxify^®^, Revance Therapeutics) was launched, the manufacturer claimed, that it could be applied transcutaneously by help of a proprietary carrier peptide derived from the HIV tat-protein. After this claim could not be substantiated, the claim was made, that the neurotoxin would stay longer at the injection site thus reducing BT spread, again by help of the proprietary peptide contained in the drug. This claim could not be substantiated neither and yet another claim was made. This time, it was that DAXI would have a longer duration of action as compared to ONA, again due to the presence of the proprietary peptide. However, the study design of the cervical dystonia registration study (Comella et al. 2024) did not include an ONA arm for comparison. Instead, the manufacturer tried a historical comparison to other BT drugs and used an endpoint definition totally different from those previously used. In the glabella line registration study (Carruthers et al. 2020), again the manufacturer did not include an ONA arm for comparison and tried a historical comparison, this time comparing high DAXI doses with lower ONA doses. Consequently, the FDA did not accept the claim of a longer duration of action of DAXI, neither for treatment of cervical dystonia nor for treatment of glabellar lines. With some surprising delay, both, the medical community and the stock markets understood these maneuvres and responded accordingly. 

For BT drug development projects this means, that marketing strategies and claims need be based on scientific data, as the medical community will scrutinise claims and contradictions and inaccuracies will be detected and may jeopardise the success of the development project.

## Summary

Developing a new BT drug is associated with several obvious challenges, including financing, manufacturing, registration and marketing. Specific challenges for BT drugs include potency, therapeutic profile, immunogenicity and product claims. While these challenges may be unexpected, addressing them is crucial for the success of the development project. Once these challenges have been properly identified and addressed, new BT drugs will generate significant opportunities more than ever before.

## Data Availability

No datasets were generated or analysed during the current study.
